# Spatial Predictions of Rhodesian Human African Trypanosomiasis (Sleeping Sickness) Prevalence in Kaberamaido and Dokolo, Two Newly Affected Districts of Uganda

**DOI:** 10.1371/journal.pntd.0000563

**Published:** 2009-12-15

**Authors:** Nicola A. Batchelor, Peter M. Atkinson, Peter W. Gething, Kim Picozzi, Eric M. Fèvre, Abbas S. L. Kakembo, Susan C. Welburn

**Affiliations:** 1 Centre for Infectious Diseases, College of Medicine and Veterinary Medicine, University of Edinburgh, Edinburgh, United Kingdom; 2 School of Geography, Highfield Campus, University of Southampton, Southampton, United Kingdom; 3 Spatial Ecology and Epidemiology Group, Department of Zoology, University of Oxford, Oxford, United Kingdom; 4 Centre for Infectious Diseases, College of Science and Engineering, University of Edinburgh, Edinburgh, United Kingdom; 5 Ministry of Health, Department of National Disease Control, Ministry of Health, Nakasero, Kampala, Uganda; New York University School of Medicine, United States of America

## Abstract

The continued northwards spread of Rhodesian sleeping sickness or Human African Trypanosomiasis (HAT) within Uganda is raising concerns of overlap with the Gambian form of the disease. Disease convergence would result in compromised diagnosis and treatment for HAT. Spatial determinants for HAT are poorly understood across small areas. This study examines the relationships between Rhodesian HAT and several environmental, climatic and social factors in two newly affected districts, Kaberamaido and Dokolo. A one-step logistic regression analysis of HAT prevalence and a two-step logistic regression method permitted separate analysis of both HAT occurrence and HAT prevalence. Both the occurrence and prevalence of HAT were negatively correlated with distance to the closest livestock market in all models. The significance of distance to the closest livestock market strongly indicates that HAT may have been introduced to this previously unaffected area via the movement of infected, untreated livestock from endemic areas. This illustrates the importance of the animal reservoir in disease transmission, and highlights the need for trypanosomiasis control in livestock and the stringent implementation of regulations requiring the treatment of cattle prior to sale at livestock markets to prevent any further spread of Rhodesian HAT within Uganda.

## Author Summary

Human African Trypanosomiasis (HAT) or sleeping sickness is a parasitic disease of humans, transmitted by the tsetse fly. There are two different forms of HAT: Rhodesian (in eastern sub-Saharan Africa), which also affects wild and domestic animals, and Gambian (in western and central sub-Saharan Africa). Diagnosis and treatment of the two diseases differ, and disease characterisation is based on prior knowledge of known geographical disease distributions. Presently, the two forms of HAT do not overlap in any area: Uganda is the only country which sustains active transmission of both types.

In recent years, Rhodesian HAT has spread into areas of Uganda that had not previously been affected, thus narrowing the gap between areas of Rhodesian and Gambian HAT transmission. This spread has raised concerns of a potential overlap of the two types of the disease, which would severely complicate their diagnosis and treatment. Earlier work indicated that Rhodesian HAT was introduced to Soroti district due to the movement of untreated cattle from affected areas. Here we show that the continued spread of HAT in Uganda (to a further 2 districts) may also have occurred due to cattle movements, despite legal requirements to treat livestock from affected areas prior to sale at markets. These findings can assist in the targeting of HAT control efforts in Uganda and show that the stringent implementation of animal treatments at livestock markets should be a priority.

## Introduction

Human African trypanosomiasis (HAT), or sleeping sickness, is caused by two sub species of a hemoflagellate parasite that are transmitted by tsetse flies. *Trypanosoma brucei rhodesiense* causes an acute disease in eastern sub-Saharan Africa and has a reservoir in wild and domestic animals while *Trypanosoma brucei gambiense* causes a chronic form of the disease in western and central sub-Saharan Africa. Uganda has had the misfortune to sustain active transmission of both types of the disease: *T. b. gambiense* in the north west and *T. b. rhodesiense* in the south east [1]. To date, however, Rhodesian and Gambian HAT have not co-existed in any area of Uganda, which is fortunate since the two forms of HAT are diagnosed and treated differently and geographical location forms the basis of diagnostic tool selection for the confirmation of diagnosis [2]. Uganda has experienced a resurgence of HAT in the past two decades. Since HAT (caused by *T. b. rhodesiense*) was introduced into Tororo District in 1987, the disease has persistently spread northwards into previously unaffected areas of Uganda [3],[4]. Since the disease imparts a considerable burden on the health systems of the poor, rural communities that it affects, the expansion of the *T. b. rhodesiense* focus is a persistent concern. The Northwards spread of disease has narrowed the area between the active foci of Rhodesian and Gambian HAT, with an estimated 150 km now separating the two forms of the disease [3]. Evidence suggests that the introduction of Rhodesian HAT into Soroti district could be attributed to the movement of untreated cattle from endemic areas through the local livestock market [5],[6]. The further spread into Kaberamaido, Dokolo, Lira and Amolotar districts raised the possibility of the potential overlap of the two types of the disease and stimulated the creation of a Public Private Partnership, Stamp Out Sleeping Sickness, to control the disease spread by treating the animal reservoir of infection [7].

It is essential that the dynamics of disease spread are understood if HAT is to be controlled in Uganda. A comprehensive understanding of the factors involved in the disease's spatial distribution and movements will enable more effective targeting of control efforts. The spatial distribution of HAT is driven by complex interactions of many factors. The occurrence of disease in an area is dependent on the establishment of disease transmission, which in turn is reliant on the suitability of an area for the disease. Within affected areas, a spatially varying intensity of transmission can result in the heterogeneous village level prevalence of disease. These two processes giving rise to i) the establishment of HAT transmission and ii) the heterogeneous prevalence of HAT in an area are likely to be driven by different environmental, climatic and social factors associated with the presence and density of tsetse flies [8]–[11], the introduction of the parasite, the presence of reservoir host species and the frequency of human-fly contact [12].

Spatial analysis and geographic information systems (GIS) have been applied increasingly to infectious disease epidemiology in recent years, including to the analysis of HAT [6], [12]–[15], animal trypanosomiasis [16]–[18] and tsetse distribution data [19],[20]. However, the factors that control the heterogeneous distribution of HAT within small areas are poorly understood, though this knowledge would be of practical use for the targeting of control efforts and the prevention of further spread. Previous studies have linked the distribution of Rhodesian HAT in Uganda with proximity to areas of swamp and low population densities [14],[15]. Distance to the local HAT treatment centre has also been found to have a confounding effect due to issues of health care accessibility [14]. In addition, several studies have examined the distribution of the tsetse fly vector, with a number of environmental variables found to have significant correlations with their distribution, including the normalised difference vegetation index (NDVI – a measure of the amount of green vegetation), humidity [21], temperature, rainfall [22] and elevation [23], utilising a variety of data sources, including remotely sensed data.

The spatial distribution of *T. b. rhodesiense* HAT in two newly affected districts of Uganda (Kaberamaido and Dokolo) was examined in relation to several environmental, climatic and social variables. Prevalence of HAT was then predicted spatially to highlight areas with the potential for high prevalence and to enable the targeting of future control efforts. The utilities of two different methodologies were compared: a two-step regression method and a traditional one-step regression method. The two-step regression was used to allow the separate analysis of factors governing the occurrence and prevalence of HAT. The prevalence analysis in the two-step regression model was conducted solely on areas that had a high predicted probability of occurrence. This was anticipated to provide an increase in predictive accuracy (for predicted prevalence) due to the exclusion of large areas with little or no HAT transmission.

## Materials and Methods

**Figure 1:**
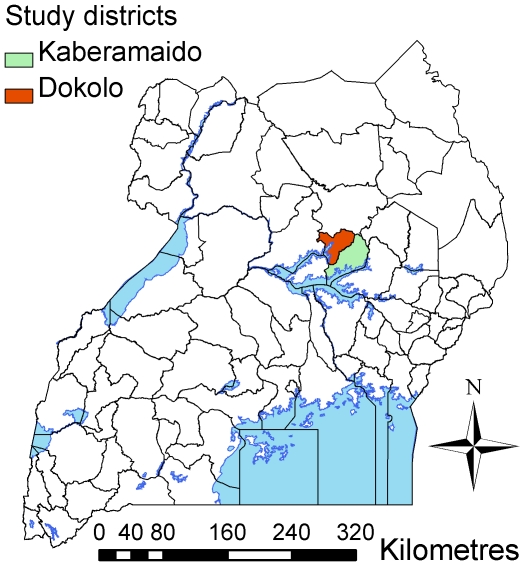
Map of Uganda highlighting study area.

The study area included Kaberamaido and Dokolo districts in Uganda (see Figure 1), two of the districts most recently affected by HAT (caused by *T. b. rhodesiense)*. Kaberamaido (Eastern region) and Dokolo (Northern region) districts lie to the north of Lake Kyoga with a combined area of approximately 2740 km^2^. The main economic activities within the study area are agriculture and fishing, with the majority of the population engaged in subsistence farming [24].

### Human African Trypanosomiasis data

A handheld global positioning system (GPS: Garmin, E-trex) was used to geo-reference the central point of all villages within the study area with guidance from local government staff. Coordinates were taken in the WGS84 geographical coordinate system in decimal degrees (data were re-projected to Universal Transverse Mercator for the calculation of distances). Comprehensive HAT hospital records were collected in collaboration with the Ugandan Ministry of Health from the two HAT treatment centres serving the study area; Lwala Hospital (Kaberamaido district) and Serere Health Centre IV (Soroti district). To maintain anonymity of subjects and patient confidentiality and to adhere to the International Ethical Guidelines for Biomedical Research Involving Human Subjects, no patient names were recorded within the database or as part of the data collection process. The hospital records were matched with the geo-referenced villages by cross-referencing each case's village of residence with the names from the geo-referenced villages. This resulted in a spatially referenced dataset of all patients residing within the study area who had received a diagnosis of HAT (normally using light microscopy).

Cases occurring from February 2004 (when the first cases were reported) to December 2006 were included in the analysis. Cases diagnosed later than December 2006 were excluded because a control programme was instigated in September 2006 that involved the mass treatment of cattle in the study area and adjoining districts. By decreasing the prevalence of human infective *T. b. rhodesiense* in the reservoir, the control programme resulted in an altered epidemiology of HAT within the study area in the subsequent year and so may have affected the results of the regression analyses.

### Covariate data

The geo-referenced HAT case data were visualised using ArcMap 9.1 (ESRI, Redlands, CA). External covariate datasets as listed in Table 1 were collected and linked with the HAT case data by village.

**Table 1:** Covariates collected for analysis, indicating variables used for model development.

Source	Variable	Spatial resolution	Units	Used in regression
	NDVI^2^ phase of annual and biannual cycle	1 km	Months	X
	Minimum and maximum NDVI^2^	1 km	No units	X
	Annual and biannual amplitude of NDVI^2^	1 km	No units	X
	Mean NDVI^2^	1 km	No units	X
Fourier processed AVHRR^1^ data sets [27]	MIR^3^ phase of annual and biannual cycle	1 km	Months	
	Minimum and maximum MIR^3^	1 km	°C	
	Annual and biannual amplitude of MIR^3^	1 km	°C	
	Mean MIR^3^	1 km	°C	
	LST^4^ phase of annual and biannual cycle	1 km	Months	X
	Minimum and maximum LST^4^	1 km	°C	X
	Annual and biannual amplitude of LST^4^	1 km	°C	X
	Mean LST^4^	1 km	°C	X
	Predicted suitability for *G. fuscipes*	1.1 km	Predicted % suitability	
Predicted tsetse suitability coverages [29]	Predicted suitability for *G. morsitans*	1.1 km	Predicted % suitability	
	Predicted suitability for *G. pallidipes*	1.1 km	Predicted % suitability	
Shuttle Radar Topography Mission [30]	Elevation	3 arc seconds	Metres	X
Landsat [28]	NDVI^2^	30 m	No units	
Landscan [31]	Population density	30 arc seconds	People per Km^2^	X
Nighttime lights of the world [32]	Nightlights	30 arc seconds	Percentage	
	Distance to gazetted land	Continuous	Kilometres	X
	Distance to river	Continuous	Kilometres	
	Distance to bush areas	Continuous	Kilometres	X
	Distance to wooded areas	Continuous	Kilometres	X
National biomass study [34]	Distance to swamp land	Continuous	Kilometres	
	Distance to permanently wet land	Continuous	Kilometres	X
	Distance to seasonally wet land	Continuous	Kilometres	X
Other geo-referenced locations	Distance to health centre (any type)	Continuous	Kilometres	X
	Distance to livestock market	Continuous	Kilometres	X

1 Advanced Very High Resolution Radiometer.

2 normalised difference vegetation index.

3 middle-infrared.

4 land surface temperature.

Several temporal Fourier-processed indices were obtained from Advanced Very High Resolution Radiometer (AVHRR) imagery: land surface temperature (LST), NDVI and middle-infrared (MIR, AVHRR channel 3). NDVI is a measure of the amount of green vegetation [25] and reflectance in the MIR band has also been linked to vegetation cover [26]. Both vegetation cover (in terms of suitable tsetse habitat) and temperature have been shown to influence the distribution of tsetse [22]. Temporal Fourier processing reduces the number of data to be processed by eliminating redundancy and characterising seasonality. The minimum, mean, maximum, phase (the timing of the cycle) and amplitude (the amount of variation around the mean) of the annual and biannual cycles were used for each of LST, NDVI and MIR. Full details regarding these data can be found in Hay et al [27]. NDVI was also calculated using the red and near-infrared wavebands of a Landsat ETM+ image (which has a finer spatial resolution than AVHRR imagery) [28] using the following formula: NDVI = (near-infrared−red)/(near-infrared + red) [25].

Predicted tsetse suitability maps were obtained from the Food and Agricultural Organization [29]. This dataset was the result of a predictive model (using tsetse fly distribution data with environmental, climatic and demographic covariates), and its reliability for the study area depends on the availability of training data from this area during the model development. Elevation [30], population density [31] and nighttime lights data [32] (which has been demonstrated to be a proxy for poverty [33]) were also obtained for use in the analyses.

Distances to physical features (in km) were calculated. Land cover data [34] were used to calculate distance to gazetted land, rivers, bush, woodland, swamps, permanently wet land and seasonally wet land. Several of these variables (bush, woodland, swamps and seasonally wet land) were the result of a quantitative interpretation of remotely sensed images along with ground data and supplementary data layers and, thus, their accuracy may be variable. These covariates were selected as potential tsetse habitats to investigate the effect of proximity of villages to these types of landcover on HAT occurrence and prevalence.

In addition, distances to the closest livestock market and health centre (of any type) were calculated using the coordinates of each of these features that were obtained during fieldwork. The distance to the closest health centre (of any type i.e. not necessarily trained or equipped to diagnose or treat HAT) was used to deal with the confounding effect of access to health care. The distance to the closest livestock market was included to investigate the possibility that cattle movements in this area may have caused or contributed to the introduction and establishment of HAT transmission, as was found in a neighbouring district [6]. The distance to the HAT treatment centre was not used as there was only one treatment centre within the study area and an additional treatment centre in the neighbouring district serving the study population, which would affect the final predictions and prevent extrapolation over a larger area. The covariates used are listed in Table 1: all were continuous variables. In addition, village population data from the most recent national census were obtained from the Uganda Bureau of Statistics [35].

### Statistical analysis

Exploratory analysis was conducted for each of the covariates: i) scatter plots to examine relationships with HAT prevalence; ii) box and whisker plots to examine the distributions of covariate data in villages which have had cases of HAT compared to villages which have not and iii) visualisation of the geographical distributions of the outcome variables in relation to the external covariates. Seventeen covariates were selected for use in the regression analyses (Table 1) based on observed relationships with HAT occurrence and prevalence and previous knowledge of significant variables from published research.

The statistical modelling was carried out using logistic regression: a generalised linear model used for the analysis of binomial data such as disease occurrence (outcome variable can take one of two possible values) or disease prevalence (where the outcome is bounded between zero and one) [36]. The modelling process describes the variability in the response variable as a function of the explanatory variables. Odds ratios (ORs) are calculated by exponentiating the regression parameters associated with each covariate; these illustrate the strength and direction of associations between the explanatory and outcome variables.

**Figure 2:**
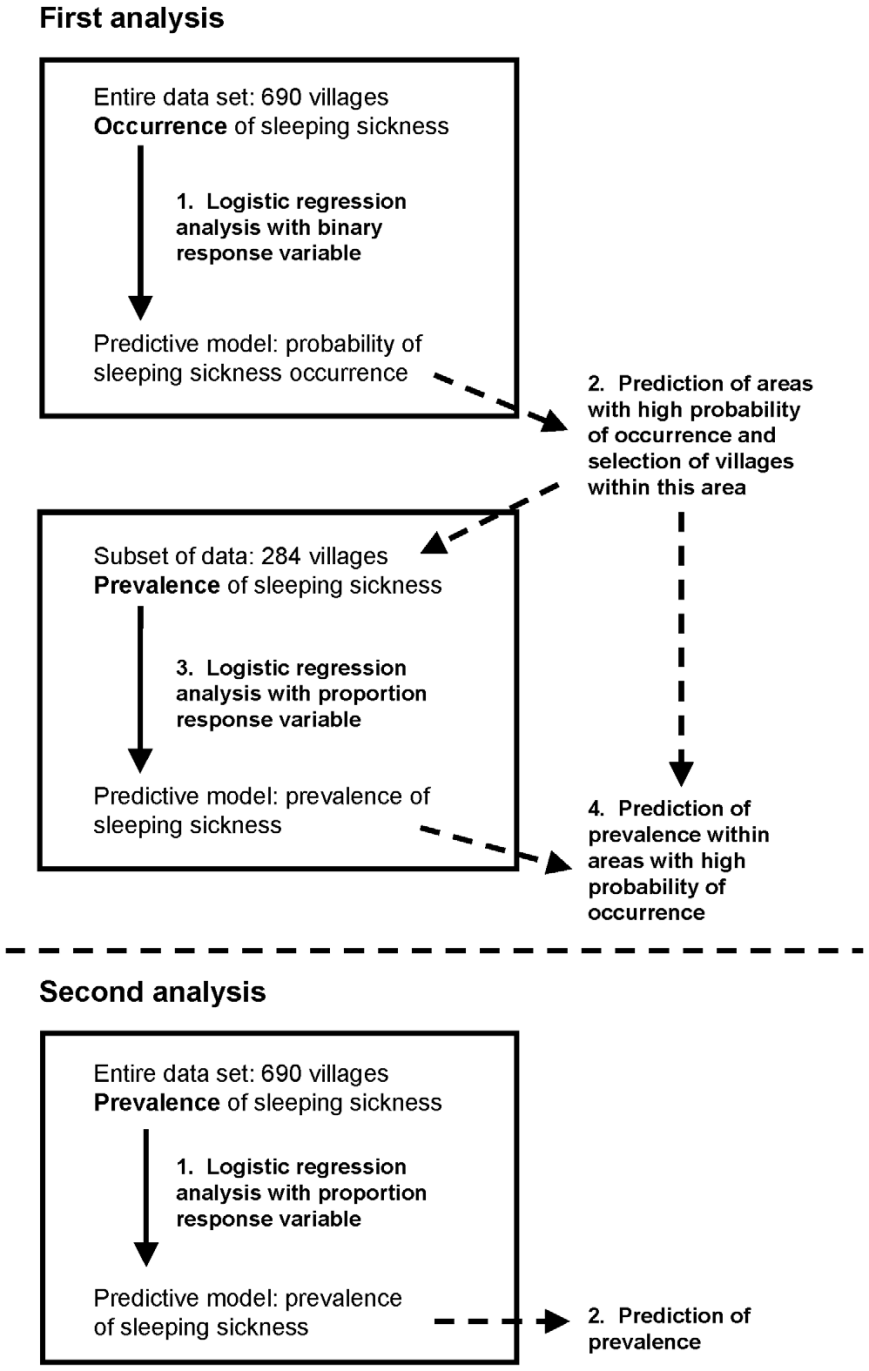
Diagram illustrating the two regression methodologies, including the main steps involved in each.

An OR of one indicates no association, an OR greater than one indicates a positive association with the odds of disease and an OR less than one indicates a negative association [36]. The size of the OR signifies the strength of the association; for example an OR of 0.5 would mean that every increase of one unit in the explanatory variable relates to a 50% reduction in the odds of disease. Likewise, an OR of 1.5 would show a 50% increase in the odds of disease for an increase of one unit for the explanatory variable. The intercept term can be interpreted as the odds of disease when all the explanatory variables are (hypothetically) zero. Statistical significance was judged at the 95% level in all analyses. All statistical analyses were carried out using R statistical software [37], and the main steps are summarised in Figure 2.

### Two-step analysis of HAT occurrence and prevalence

This methodology comprised two logistic regression models applied sequentially (first analysis, Figure 2). An initial model was fitted that predicted probability of HAT occurrence using the HAT status of all villages in the study area as the outcome of interest. Villages for which at least one case of HAT was reported during the study period were classified as case villages, while villages for which no cases were reported were treated as controls (giving a binary outcome).

The two-step model was developed to test its predictive capability against a traditional regression analysis and to investigate aspects of the underlying epidemiology affecting the spatial heterogeneity in disease occurrence (which villages had been affected by HAT) as well as prevalence (how intense was the transmission within affected areas) which are confounded in a one-step approach.

Forwards stepwise addition beginning with the null model (no explanatory variables) was used in the model fitting. At each step the variable resulting in the greatest reduction in deviance was selected. A Chi-squared likelihood ratio test was used to compare models, and additional explanatory variables were accepted only if this test was significant and the covariate was significant within the model. Any variables that lost significance in subsequent steps were removed from the model. The stepwise addition of plausible interaction terms (if interaction is present the effect of one variable on odds of disease changes in relation to the effect of another variable) was then carried out in the same manner after the variables were centred (variable mean was subtracted from each value).

The sensitivity (true positive rate) and specificity (true negative rate) of the fitted model were calculated for a variety of cut-off points (the value of the predicted probability of occurrence above which a location would be defined as a case village) using the predicted and observed values, and plotted against the cut-off points. The cut-off point where the sensitivity and specificity crossed was selected as a suitable cut-off point for the classification of case and non-case villages: this point maximises both the specificity and the sensitivity of the classification of locations. A 10-fold cross-validation (where predicted values are compared with observed values) was performed using ten random sub-divisions of the dataset. The area under the receiver operator characteristic curve (AUC) was calculated; this value gives a measure of the overall performance of the model in classifying villages. An AUC of 1 indicates perfect discrimination between case and control villages, and an AUC of 0.5 illustrates a model that is in effect worthless for discrimination purposes.

The resulting regression equation (probability of occurrence as a function of the explanatory variables) was used to predict probability of occurrence of HAT across a grid with an area of 30,000 km^2^ (including the study region) and a 1.1 km cell size (this was the minimum spatial resolution from the covariate datasets). All villages within the study area lying within an area of high predicted probability of occurrence (probability of occurrence above the selected cut-off value) were extracted for use in the second step of the analysis.

The outcome variable for the second step of the two-step regression was defined as prevalence of HAT (number of cases divided by village population). Prevalence data from all villages within areas of high predicted probability of occurrence were included in the model, including those with no reported cases (i.e. a reported prevalence of zero). Forwards stepwise addition was used in the model fitting procedure, as for the first step. For this section of the analysis, the distance to health centre variable was forced into the model (regardless of it's significance) to ensure that access to health care was controlled for in the final results. The fitted model was used to predict the prevalence of Rhodesian HAT across the same area as was used in the first step.

### One-step analysis of prevalence using all villages

For the one-step analysis (second analysis, Figure 2), the same methodology was used as the second step of the two-step regression, using prevalence data from all villages.

## Results

**Figure 3:**
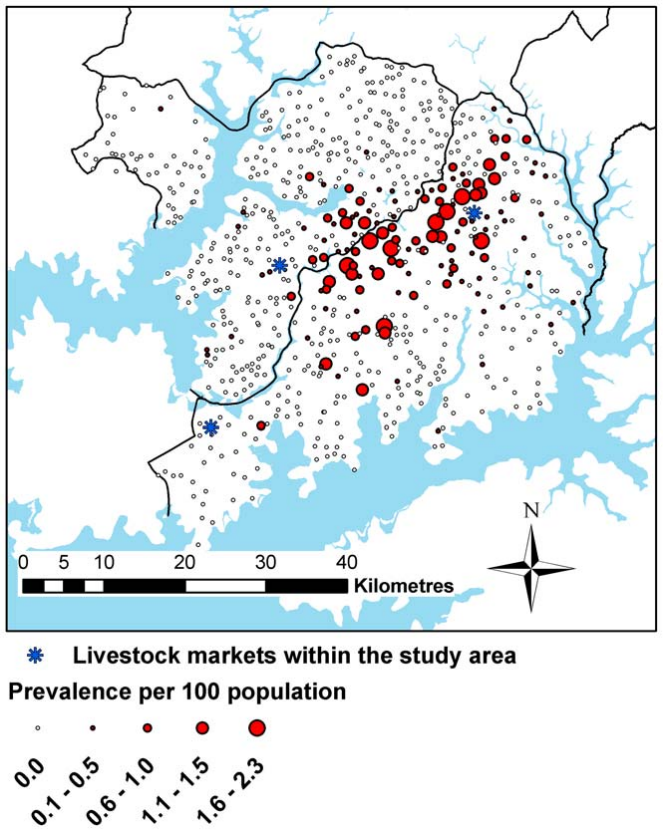
Village level period prevalence of HAT, 2004–2006. Blue areas represent water bodies. District boundaries are also shown as black lines.

A total of 690 villages within Kaberamaido and Dokolo districts were geo-referenced. Two villages were not geo-referenced due to logistical difficulties, and 18 villages that had recently separated into two were merged for the purpose of the analysis. A total of 52 patient records could not be matched to any of the known villages in the study area and so were excluded from the analysis. This was most likely due to inaccuracies in the recording of patient details in the hospital records. The total number of cases used in the study was 302. The distribution of villages, along with the village prevalence of HAT using data from 2004–2006 is illustrated in Figure 3.

### Two-step regression analysis of HAT suitability and prevalence

Four covariates were found to influence significantly the occurrence of HAT across the study area (*p*<0.05) as shown in Table 2. Occurrence of HAT was negatively correlated with distance to the closest livestock market, with a 21% reduction in odds of disease for every kilometre increase in distance when accounting for the additional variables. This was found to interact (the effect of one variable on odds of disease changes in relation to the effect of another variable) with maximum NDVI, which also demonstrated a negative correlation with HAT occurrence. In addition, occurrence was positively correlated with minimum LST and negatively correlated with distance to the closest health centre.

**Table 2:** Results of the first model from the two-step regression analysis, using a binary response variable and all villages.

Variable	Odds ratio (95% CI)	p-value
Intercept	7.42E−6 (3.39 E−8–0.002)	<0.0001
Distance to livestock market	0.79 (0.75–0.84)	<0.0001
Maximum NDVI	9.56 E−7 (2.64 E−12–0.35)	0.03
Minimum LST	2.10 (1.44–3.04)	0.0001
Distance to health centre	0.84 (0.74–0.94)	0.002
Distance to market * Max NDVI	32.46 (3.34–315.52)	0.003

**Figure 4:**
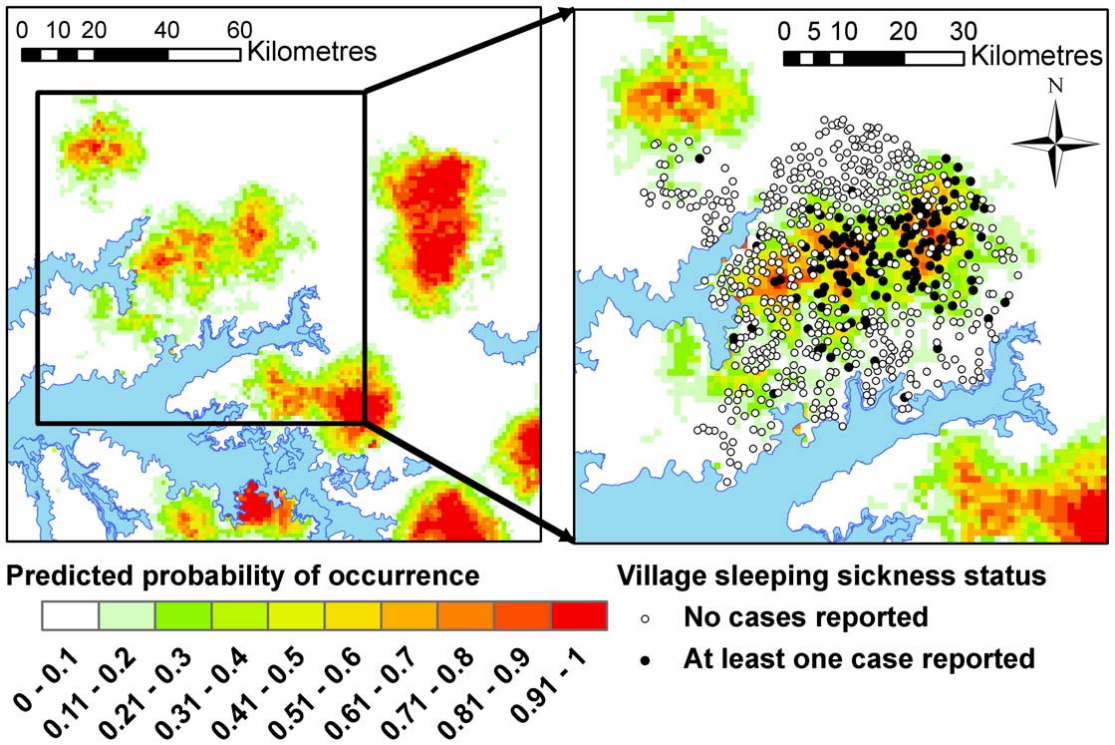
Predicted probability of HAT occurrence from the first step of the second analysis. White and pale green indicate areas with low predicted probability of occurrence. Black circles indicate case villages and white circles represent non-case villages within the study area.

For prediction purposes, the selected probability cut-off point for the prediction of areas suitable for transmission was 0.2, and model diagnostics indicated that the model provided a reasonable fit to the data, and reliable predictions (AUC: 0.87, 10-fold cross-validation estimate of accuracy: 85%). The predicted suitability for transmission across the study area using the specified model is illustrated in Figure 4.

The prediction was used to create a mask over the study area; all areas with a predicted probability of occurrence less than 0.2 were excluded. 279 villages lay within the area defined as having a high probability of occurrence. However, seven of those villages had no population data and so were excluded from the remaining analysis leaving 272 villages. The results from the second (prevalence) model are shown in Table 3.

**Table 3:** Results of the second step from the two-step regression analysis, using prevalence response variable and a subset of villages.

Variable	Odds ratio (95% CI)	p-value
Intercept	1.72 E−8 (1.78 E−12–0.0002)	0.0001
Distance to health centre^1^	0.92 (0.85–1.00)	0.05
Distance to livestock market	0.80 (0.77–0.83)	<0.0001
NDVI phase of annual cycle	3.46 (1.67–7.14)	0.0008
NDVI annual amplitude	2.18 E+11 (1.85 E+6–2.59 E+16)	<0.0001
LST phase of annual cycle	1.27 (1.13–1.43)	<0.0001
Distance to woodland	1.15 (0.95–1.40)	0.18
Distance to bush	0.93 (0.90–0.97)	0.0007
Maximum NDVI	3.50 E−5 (1.46 E−8–0.08)	0.01
LST annual amplitude	1.27 (1.07–1.52)	0.009
Minimum LST	1.46 (1.13–1.89)	0.004
Distance to livestock market * Distance to woodland	0.91 (0.86–0.97)	0.002

1 Forced into the model to ensure that access to health services was controlled for in the model.

HAT prevalence was significantly correlated with nine variables in addition to distance to the closest health centre that was negatively correlated and of borderline significance (*p* = 0.05, variable forced into the model). Prevalence was negatively correlated with distance to the closest livestock market with every additional kilometre resulting in a 20% decrease in odds of disease. This was shown to interact with distance to the closest area of woodland, which in turn showed a positive correlation with prevalence. In addition, HAT prevalence was negatively correlated with distance to the closest area of bush and maximum NDVI and positively correlated with NDVI phase of annual cycle, NDVI annual amplitude, LST phase of annual cycle, LST annual amplitude and minimum LST.

**Figure 5:**
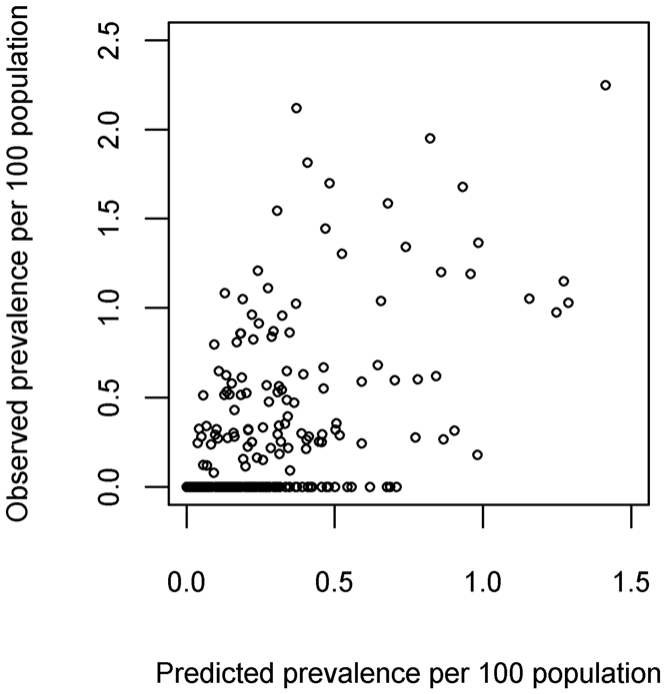
Scatter plot of observed prevalence versus predicted prevalence (per 100 population) using the two-step analysis.

**Figure 6:**
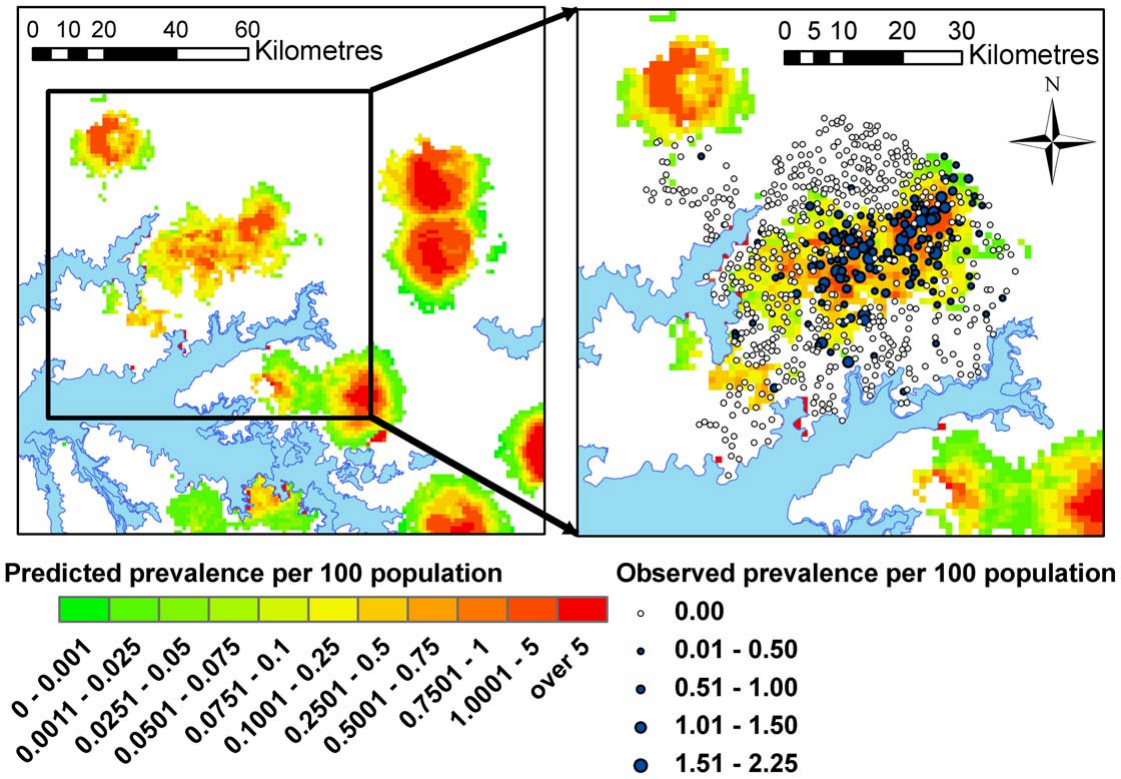
Predicted prevalence of HAT from the second step of the two-step analysis. White indicates areas predicted to be unsuitable for transmission. Blue circles indicate case villages and white circles represent control villages within the study area, with increasing circle size denoting increasing village period prevalence (2004–2006).

The two-step regression analysis resulted in a correlation between observed and predicted prevalence of 0.57 (a value of 1 indicates perfect correlation and 0 no correlation). The model had a small tendency to over predict prevalence with a median error of 0.05% (error calculations are based on prevalence per 100 population and so are expressed as a percentage). The mean absolute error for the predicted prevalence per 100 population was 0.24%. The scatter plot of predicted prevalence against observed prevalence (Figure 5) shows a tendency for over-prediction of prevalence in villages with an observed prevalence of zero. The predicted prevalence from the two-step analysis is shown in Figure 6.

### One-step regression analysis of prevalence using all villages

Nine variables were shown to be significantly associated with prevalence of HAT across the study area using the one-step regression, as shown in Table 4. HAT prevalence was negatively correlated with distance to the closest livestock market with a 21% reduction in odds of disease for every kilometre increase in distance. This was shown to interact significantly with both NDVI phase of annual cycle and distance to the closest area of woodland, both of which were also negatively correlated with prevalence. Additionally, prevalence was negatively correlated with maximum NDVI, mean LST and distance to the closest health centre. HAT prevalence was positively correlated with minimum LST, LST phase of annual cycle and LST annual amplitude.

**Table 4:** Results of one-step regression analysis using prevalence outcome variable and all villages.

Variable	Odds ratio (95% CI)	p-value
Intercept	0.32 (0.0005–200.1)	0.003
Distance to livestock market	0.79 (0.76–0.82)	<0.001
Maximum NDVI	2.6E−06 (3.59 E−9–0.002)	0.0001
Minimum LST	2.05 (1.62–2.60)	<0.0001
LST phase of annual cycle	1.26 (1.12–1.42)	<0.0001
LST annual amplitude	1.75 (1.36–2.26)	<0.001
Mean LST	0.56 (0.39–0.81)	0.003
Distance to woodland	0.96 (0.76–1.22)	0.76
Distance to health centre	0.87 (0.80–0.94)	<0.0001
NDVI phase of annual cycle	0.98 (0.42–2.33)	0.97
Distance to market * NDVI phase of annual cycle	0.84 (0.74–0.94)	0.002
Distance to market * distance to woodland	0.95 (0.91–0.99)	0.01

**Figure 7:**
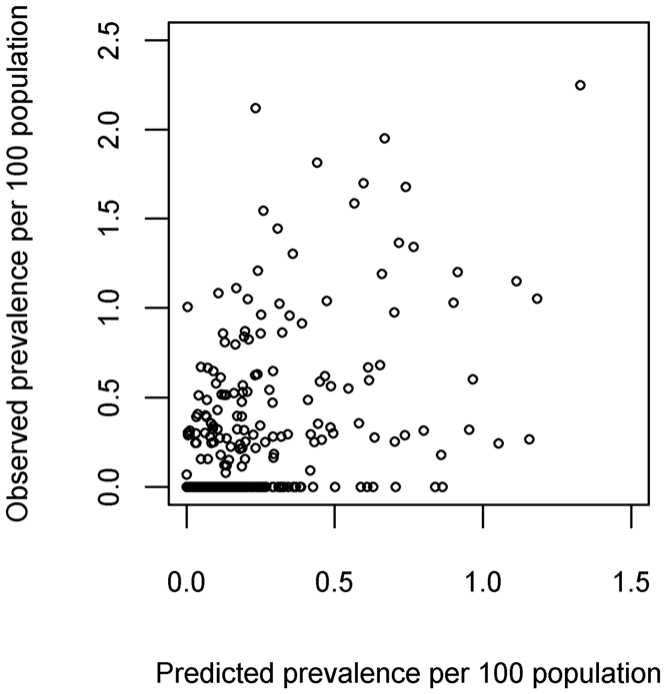
Scatter plot of observed prevalence versus predicted prevalence (per 100 population) using the one-step analysis.

**Figure 8:**
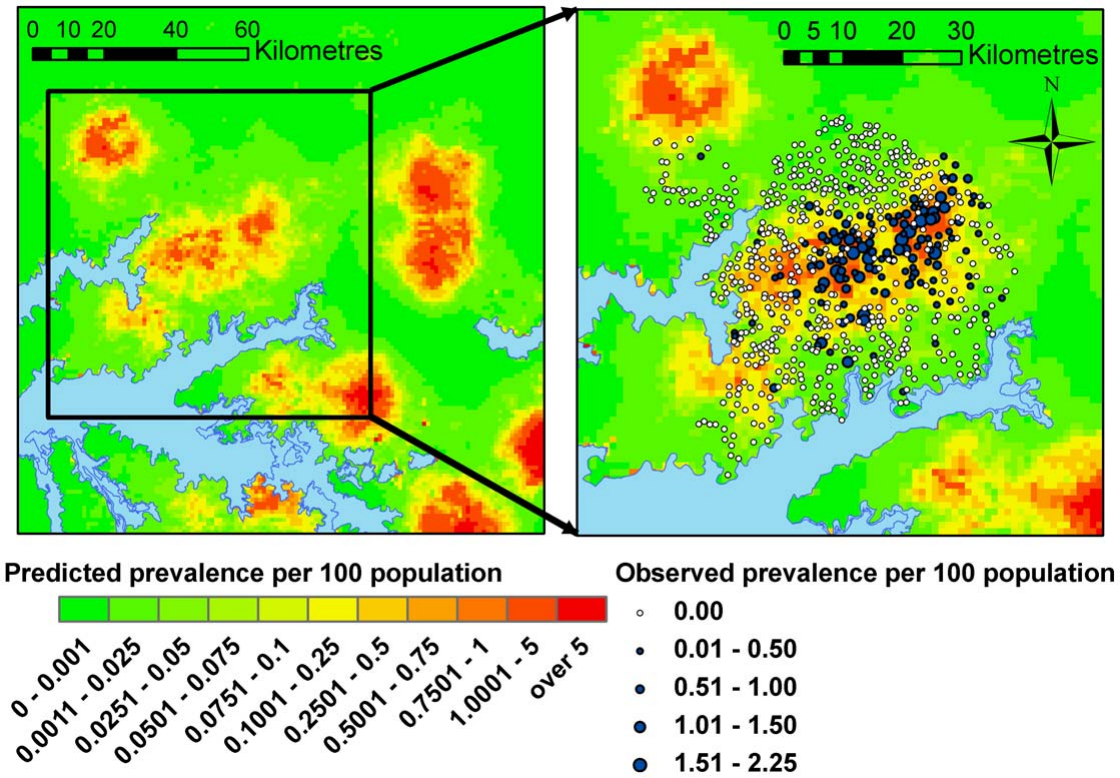
Predicted prevalence of HAT from one-step regression analysis.

The correlation between predicted and observed prevalence values was 0.58 indicating a modest linear association. The model was slightly biased with a very small tendency to over-predict prevalence (median error = 0.02%) and the mean absolute error was 0.13% (calculated based on prevalence per 100 population and so expressed as a percentage). The scatter plot of predicted prevalence against observed prevalence values (Figure 7) illustrates that many of the errors are associated with over-prediction for villages with observed prevalence of zero. Figure 8 shows the predicted prevalence across the study area using the final prevalence model.

To allow a direct comparison of the predictive accuracy of the two methodologies, the one-step model was used to calculate predicted prevalence for the villages with high predicted probabilities of occurrence from the two-step analysis (i.e. excluding areas with a predicted probability of occurrence of less than 0.2). The correlation between predicted and observed prevalence was 0.50, lower than that for the two-step regression method (0.57). Again, the model was shown to have a tendency to over predict prevalence, with a median error of 0.05% (calculated using prevalence per 100 population). The mean absolute error was 0.24%, equal to the mean absolute error from the two-step regression methodology.

## Discussion

Spatial determinants for HAT are poorly understood across small areas. This study examined the relationships between Rhodesian HAT and several environmental, climatic and social factors in two newly affected districts, Kaberamaido and Dokolo. The application of a two-step regression approach for the prediction of HAT prevalence in a newly affected area of Uganda allowed the investigation of factors influencing the occurrence and prevalence of HAT separately, and overall resulted in a slight increase in predictive accuracy when compared to a one-step analysis in areas with high predicted probability of occurrence. Each of the models has illustrated an increased risk of HAT in villages closer to livestock markets than in villages further away, suggesting the persistent spread of Rhodesian HAT in Uganda may have resulted from the continued movement of untreated cattle.

**Figure 9:**
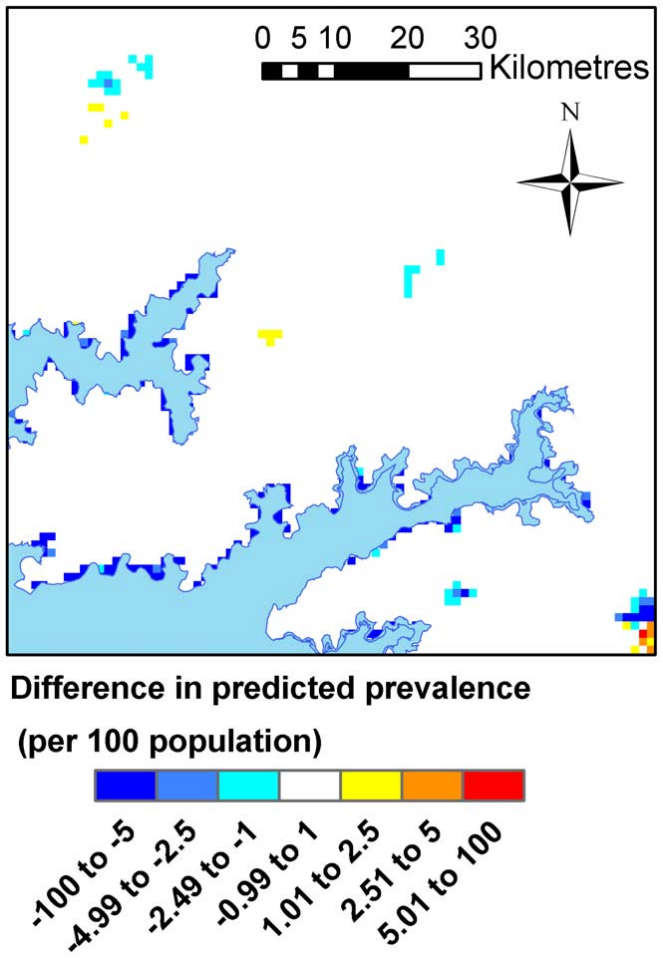
Difference in predicted prevalence between first and second analysis.

The two-step regression model gave a slight increase in predictive accuracy in comparison with the one-step analysis with a correlation between fitted and observed prevalence values of 0.57 for the two-step regression and 0.50 for the one-step regression analysis (when looking only at areas with a high predicted probability of occurrence). Both models tended to predict higher prevalence than was observed, particularly in villages of zero prevalence, with a median error of 0.05% for both models. The mean absolute error was equal for the two methods (0.24%). The difference in predicted prevalence of HAT from the two methods was small over the majority of the prediction area, with divergences mainly occurring in areas of high predicted prevalence outside of the study area (see Figure 9).

There were only two health centres trained and equipped to diagnose and treat HAT serving the study population during the study period. It has been shown previously that levels of geographical accessibility to treatment facilities can have an effect on the observed spatial distribution of HAT, with smaller numbers of cases reported from areas which are further from the treatment centres [13]. However, an added complication arises in this study as the choice of site for the main HAT treatment facility in the area (at Lwala Hospital) was driven in part by its location within the focus of new cases of HAT in 2004. Moreover, this facility is close to one of the major livestock markets in the study area (7.5 km away) making their separate influences on observed prevalence difficult to distinguish.

Distance to the closest livestock market was an important predictor in the one-step regression and in both steps of the two-step regression, with decreasing odds of infection at increasing distances. Previous research has confirmed the introduction of HAT to a previously unaffected area via the introduction of untreated, infected livestock [6]. These results suggest that despite reinforced policy regarding the treatment of livestock for trypanosomes prior to movement from endemic areas [38], the ongoing spread of HAT into Kaberamaido and Dokolo may have been facilitated by the movement of infected cattle through one or more of the local livestock markets. The main cattle trading routes within this part of Uganda run from *T. b. rhodesiense* endemic areas in the south east, through the study area and neighbouring districts, to the *T. b. gambiense* endemic areas in the far north west of Uganda towards southern Sudan. Clearly, this increases the risk of overlap of the two subspecies, particularly if the regulations regarding the treatment of cattle being moved from *T. b. rhodesiense* endemic areas continue to be broken. The stringent implementation of regulations requiring the treatment of cattle prior to sale at livestock markets should be a priority for the Ugandan Government and tsetse control efforts may be more efficiently targeted to areas surrounding livestock markets to prevent the establishment of transmission in previously unaffected areas as occurred in Soroti district in the late 1990s and Kaberamaido and Dokolo districts in 2004.

Other variables that were also significantly correlated with HAT prevalence and/or occurrence included distance to the nearest health centre, maximum Normalised Difference Vegetation Index (NDVI), NDVI phase of annual variation, NDVI annual amplitude, minimum Land Surface Temperature (LST), LST phase of annual variation, LST annual amplitude, mean LST, distance to the closest area of woodland and distance to the closest area of bush. The significance of these variables highlights the importance of climatic and environmental conditions for HAT transmission. Distance to the closest health centre was also a significant factor in each model, with decreasing prevalence observed at increasing distances. This suggests a confounding relationship due to accessibility of health services as has been previously reported [13].

Each of the regression models (the one-step regression model and each step of the two-step regression models) included maximum NDVI (negative association) and minimum LST (positive association) as significant predictors. These are likely to relate to the habitat and environmental requirements of the tsetse fly vector of disease. The additional variables found to be significantly correlated with HAT prevalence in each analysis are probably also linked to the suitability of an area for the tsetse fly vector (due to their preferred habitat and also climatic requirements), and so will influence the intensity of transmission and observed prevalence of HAT.

Analysis of the residual variation (after accounting for the covariate effects) indicated that there was some spatial autocorrelation in the residuals from the one-step regression and the probability of occurrence analysis (first step of the two-step regression analysis). For the two-step regression, the probability of occurrence regression was carried out partially to provide a mask over areas with low predicted probability of occurrence to enable the focusing of the prevalence analysis, and so the small amount of spatial autocorrelation in the residuals is not seen as problematic as it would have a negligible effect on the final prevalence model. However, for the one-step regression, the small amount of spatial autocorrelation in the residuals may lead to inflated statistical significance for some of the covariates. Further research is underway to address this autocorrelation in the residuals and to assess any increase in the predictive accuracy using a model-based geostatistics approach [39].

From these and previous findings [6], it is thought to be likely that the movement of *T. b. rhodesiense* infected livestock from endemic areas through livestock markets within the study area occurs periodically. A complex interaction of factors is involved in the establishment of transmission following such an occurrence. In addition to the variables included in the current analysis, tsetse and livestock densities, human-cattle-tsetse contact and also to a large degree, chance, may play roles. Further research is planned to build upon these findings, incorporating detailed livestock market data and cattle trading networks to give a more thorough understanding of the spatial and temporal dynamics of HAT within Uganda.
